# Ultra-late triple metastasis to the breast, lung, and brain 25 years after nephrectomy for clear cell renal cell carcinoma: a case report and literature review

**DOI:** 10.3389/fonc.2026.1733997

**Published:** 2026-01-21

**Authors:** Sujian Xiao, Xinyu Wang, Xiaoling Deng, Xin Huang, Chuance Du, Huozhong Yuan, Yanyan Li, Qiliang Zhai

**Affiliations:** 1 Department of Breast, The Affiliated Ganzhou Hospital, Jiangxi Medical College, Nanchang University, Jiangxi, China; 2 Department of Urology, The Affiliated Ganzhou Hospital, Jiangxi Medical College, Nanchang University, Jiangxi, China; 3 Infectious Disease Department, The First Affiliated Hospital, Jiangxi Medical College, Nanchang University, Nanchang, Jiangxi, China; 4 Pathology Department, The Affiliated Ganzhou Hospital, Jiangxi Medical College, Nanchang University, Jiangxi, China

**Keywords:** breast metastasis, case report, clear cell renal cell carcinoma, immunotherapy, lifelong follow-up, ultra-late recurrence

## Abstract

**Background:**

Clear cell renal cell carcinoma (ccRCC) most commonly metastasizes to the lungs, lymph nodes, bones, and liver. Metastasis to the breast is exceptionally rare, representing less than 0.5% of cases. Herein, we report an unprecedented case of triple metastasis with the longest recorded latency period.

**Case presentation:**

An 86-year-old female presented with a painless left breast mass 25 years after undergoing radical nephrectomy for ccRCC. Multi-modality imaging (mammography, ultrasound, MRI) revealed a suspicious breast lesion. Subsequent chest CT and PET-CT identified synchronous metastatic nodules in the lungs and brain. Histopathological examination of the resected breast mass confirmed metastatic carcinoma. The diagnosis of ccRCC metastasis was substantiated by an immunohistochemical profile positive for PAX8, CAIX, and CD10, while negative for estrogen receptor, progesterone receptor, and HER2.

**Conclusion:**

This case highlights breast metastasis as a rare but critical differential diagnosis in patients with a history of ccRCC, even decades after initial treatment. It underscores the necessity of lifelong follow-up for ccRCC survivors and demonstrates the integral role of imaging and pathological confirmation in guiding clinical diagnosis. Furthermore, the remarkable 25-year latency period challenges existing surveillance paradigms and provides a compelling rationale for the use of combined immunotherapy and targeted agents in managing ultra-late, multi-metastatic recurrences.

## 1 Introduction

Renal cell carcinoma (RCC) is a heterogeneous malignancy with a broad spectrum of clinical manifestations and histological subtypes (1). Despite surgical treatment, nearly 40% of patients with localized RCC may develop distant metastases (2). Metastasis to the breast, however, is an exceedingly rare phenomenon in RCC (3). We report a case of triple metastasis to the brain, lungs, and breast with an exceptional latency period of 25 years following nephrectomy.

This case represents one of the longest metastatic latencies (25 years) reported in the literature for renal clear cell carcinoma with multiple rare metastases.

## 2 Case presentation

This patient was 86 years old and had undergone surgery 26 years ago for a right kidney tumor. The histopathological diagnosis of the surgical resection specimen was clear cell renal cell carcinoma.

The patient presented to our department for evaluation of a painless breast mass that was first noticed approximately one year earlier and underwent a physical examination. In the lower inner quadrant of the left breast, at the 8 o’clock position, a mass about 2 cm × 1.5 cm in size could be felt, 4 cm away from the areola. The mass was hard, had good mobility, did not adhere to the skin, and there were no abnormalities in the surrounding skin. No obvious enlarged lymph nodes were found in the ipsilateral axilla and supraclavicular area. No obvious abnormalities were detected in the right breast.

**Figure 1:**
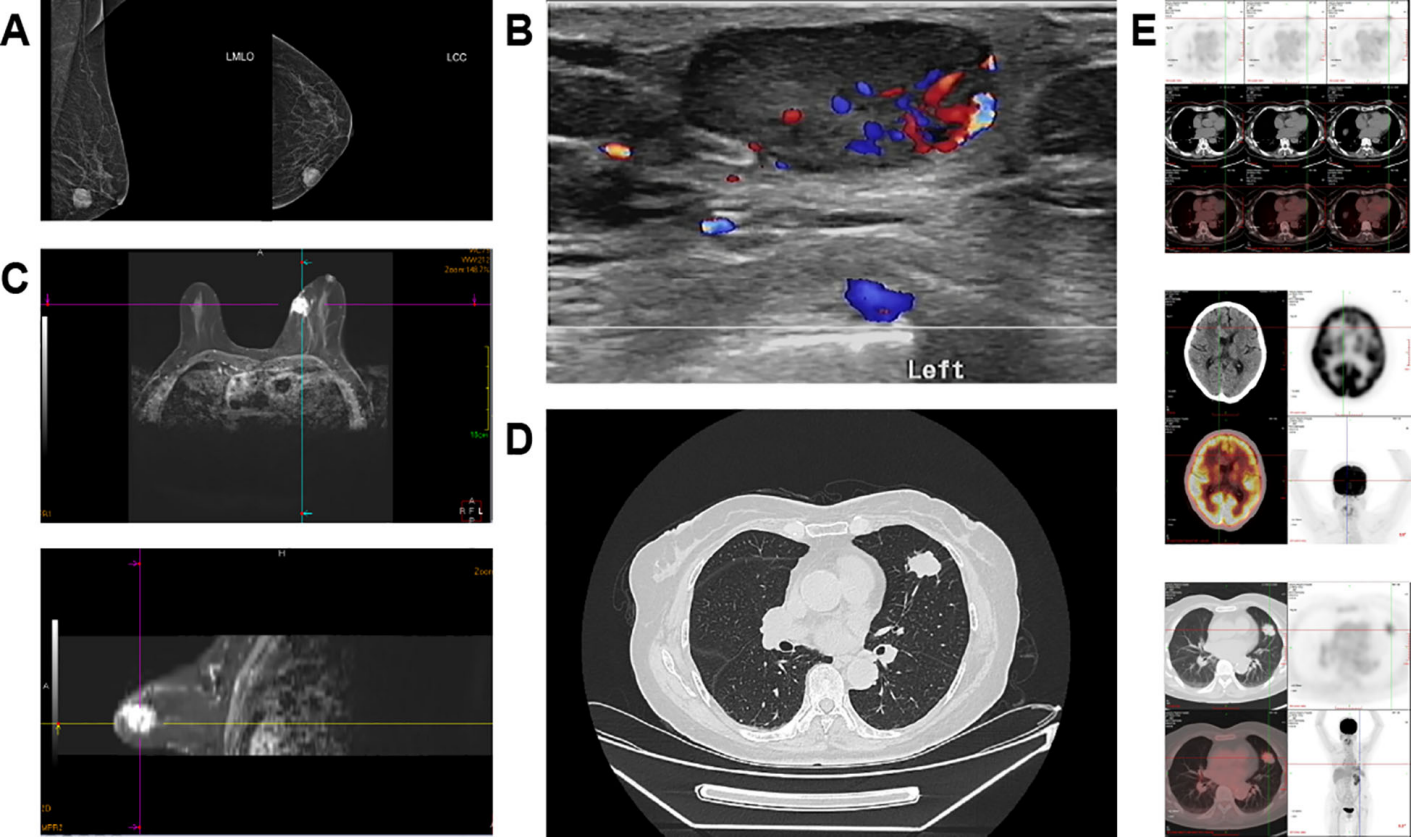
The imaging examination results of the patient. **(A)** Mammography of the breast. **(B)** Breast magnetic resonance imaging (MRI). **(C)** Color Doppler ultrasound of the breast. **(D)** Thoracic-abdominal computed tomography (CT) at the level of the lungs. **(E)** Positron emission tomography/computed tomography (PET/CT).

Upon admission, mammography (**Figure 1A**) revealed a round, homogeneous mass in the lower inner quadrant of the left breast, located in the middle third of the subcutaneous tissue, measuring approximately 18.4 mm × 16.4 mm × 17.9 mm. The mass had well-defined borders, with no abnormal calcifications or structural distortions. The skin of both breasts was normal, with no signs of thickening, nipple retraction, or areolar abnormalities. The subcutaneous fat layer was clear, and no thickening or traction of the suspensory ligament was observed. No abnormal lymph nodes were found in the anterior axillae.

Breast ultrasound (**Figure 1B**) identified a non-regularly shaped mass in the same location, measuring approximately 20 mm × 12 mm. The mass exhibited uneven edges, clear boundaries, and low internal echoes. No calcifications were detected, and the posterior echo was unremarkable. Color Doppler flow imaging (CDFI) revealed rich blood flow within the mass.

Breast MRI (**Figure 1C**) showed a circular-like nodule in the inner quadrant of the left breast, with slightly short T1 and long T2 abnormal signals. Diffusion-weighted imaging (DWI) revealed high signals, while the apparent diffusion coefficient (ADC) showed isointense to hyperintense. The lesion size was approximately 2.3 cm × 2.2 cm × 2.0 cm. After gadolinium-DTPA enhancement, the lesion exhibited significant enhancement with clear boundaries, and the time-signal curve displayed an “outflow” pattern.

Chest CT (**Figure 1D**) identified a solid nodule in the upper lingular segment of the left lung, measuring approximately 28.4 mm × 22.6 mm, as well as a second nodule in the right upper lobe, measuring approximately 10.1 mm × 7.5 mm. These findings raised concern for metastasis, prompting PET-CT imaging for further evaluation(**Figure 1E**). The PET-CT findings are highly suggestive of concurrent metastatic involvement in the brain and lungs.

**Figure 2:**
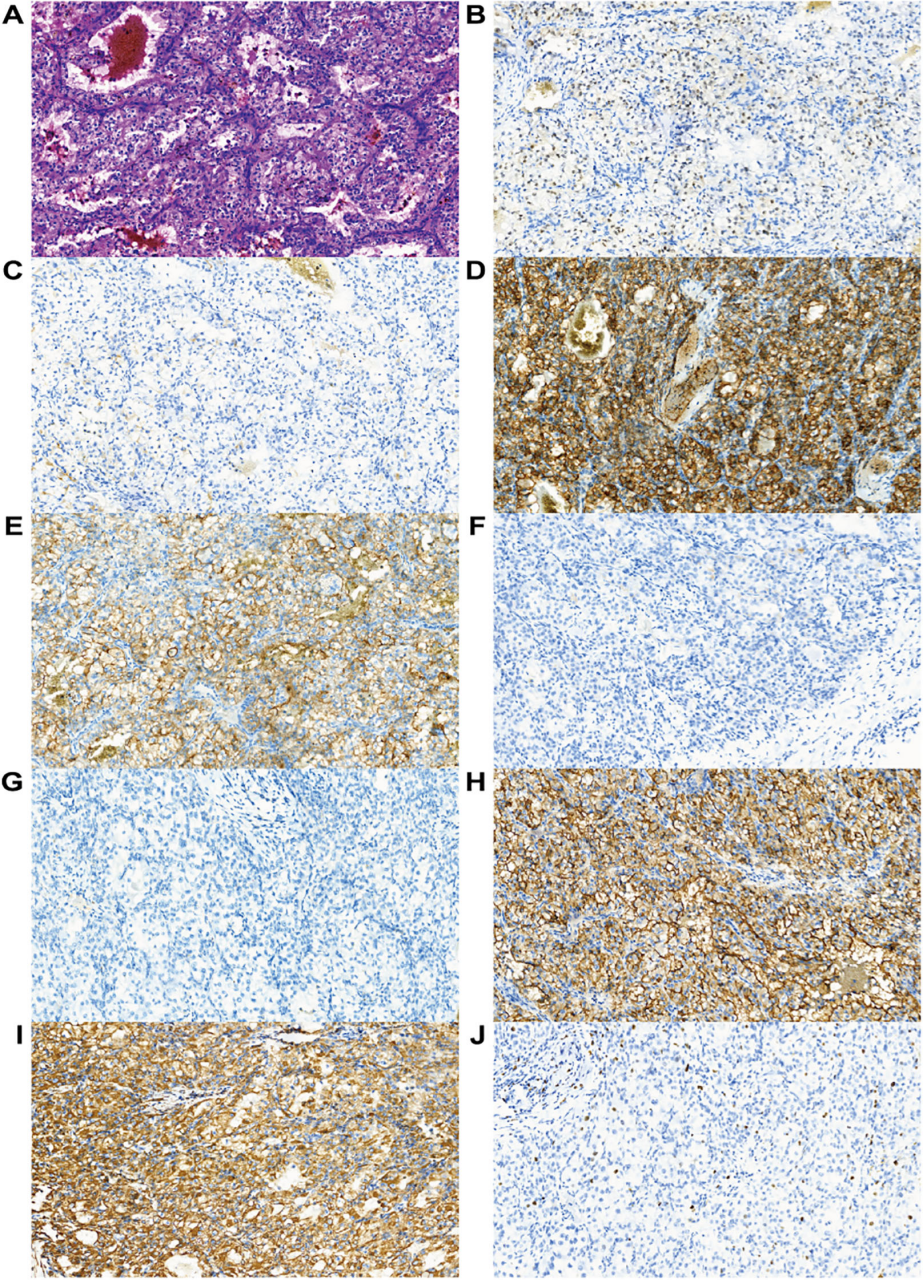
Histological examination. **(A)** Hematoxylin and eosin staining (HE) (magnification, ×200). Immunohistochemical staining for **(B)** PAX8 (partially positive; magnification, ×200), **(C)** CAM5.2 (partially positive; magnification, ×200), **(D)** CA9 (positive; magnification, ×200), **(E)** CD10 (positive; magnification, ×200), **(F)** CK (negative; magnification, ×200), **(G)** INSM1 (negative; magnification, ×200), **(H)** EMA (positive; magnification, ×200), **(I)** Vimentin (positive; magnification, ×200), and **(J)** Ki-67 (positive in 4% of tumor cells; magnification, ×200).

Given the patient’s history of pulmonary and cerebral metastases, a lumpectomy of the left breast mass was performed under local anesthesia. Postoperative pathology (**Figure 2**) confirmed metastatic carcinoma in the breast. Based on the clinical presentation and immunohistochemical profile, the tumor was most likely of renal origin. The immunohistochemical results were as follows: CK (-), Ki67 (4%+), INSM1(-), CAM5.2 (partial+), EMA(+), PAX8 (partial+), CAIX(+), CD10(+), Vimentin(+).

## 3 Discussion

### 3.1 Distribution of metastases in clear cell renal cell carcinoma

Clear cell renal cell carcinoma (ccRCC) is the most common subtype of renal cell carcinoma, accounting for 70%–80% of all renal cancer cases. Metastases from ccRCC typically occur in the lungs (70%), followed by lymph nodes, bones, and liver (4). In contrast, breast metastases are extremely rare (5) (**Table 1**). This case represents an exceedingly rare instance of triple metastasis to the brain, lungs, and, notably, the breast, following the resection of ccRCC. The mechanisms underlying such extraordinary dormancy remain elusive but may involve immune-mediated equilibrium and angiogenic incompetence, which were eventually overcome by yet unidentified factors. Building on this, immunosenescence, the age-related decline in immune function, plays a critical role in disrupting this equilibrium. As individuals age, adaptive immune responses, particularly T-cell functionality, weaken, leading to reduced surveillance of dormant cancer cells and increased susceptibility to tumor progression (6). This process is compounded by the accumulation of senescent immune cells in the tumor microenvironment, which can impair anti-tumor immunity and correlate with poorer prognosis (7). Furthermore, a shift toward a pro-inflammatory microenvironment may act as a key trigger, where chronic inflammation—driven by cytokines, chemokines, and inflammatory bodies—creates a permissive niche for dormant cells to reactivate and metastasize (8). In the context of late metastases in RCC, this inflammatory TME can synergize with angiogenesis and metabolic changes, facilitating the “awakening” of disseminated tumor cells after decades of dormancy (9). From an academic perspective, this case of exceptionally prolonged latency may represent a classic exemplar of the “early dissemination-late metastasis” paradigm. This model postulates that, prior to primary tumor resection, cancer cells may have already seeded distant organs, entering a long-term dormant state potentially maintained by a combination of therapeutic pressures and host immune surveillance. Ultimately, the “awakening” of these dormant cells and the emergence of clinical metastases may be triggered by a shift in this equilibrium, potentially driven by age-related immunosenescence or the development of a pro-inflammatory microenvironment. Clinically, breast metastases often present as unilateral or bilateral painless breast masses, which are frequently misdiagnosed as primary breast cancer or other breast diseases (10). Due to similarities in imaging and pathological features between metastatic ccRCC and primary breast cancer, diagnosis and treatment pose significant challenges (10, 11).

**Table 1:** Distribution of metastasis in renal clear cell carcinoma (4, 5).

Transferred site	Percentage (%)
Lung	70%
Lymph node Bone	45%
Liver	32%
Adrenal gland	18%
Brain	10%
Pancreas	8%
Pleura	5%
Peritoneum	4%
Spleen Thyroid gland	2%
Intestine	0.9%
Thyroid	0.7%
Bowel	0.7%
Breast	0.3%

Although the probability of brain metastasis in ccRCC can be as high as 8%, the co-occurrence of breast and brain metastases remains low. Moreover, the prognosis for ccRCC with brain metastasis is poorer compared to metastases in other sites, with a median survival of 16.5 months, which is significantly lower than that of “low-risk” metastatic sites such as the pancreas (50.1 months) and thyroid (44.0 months). The blood-brain barrier may further limit drug delivery and impair treatment efficacy.

In recent years, advancements in molecular biology and imaging technology have improved the diagnosis of breast metastases from ccRCC. ccRCC can metastasize to the breast many years or even decades after surgery (12, 13). One reported case described breast metastasis occurring 18 years after nephrectomy (5). The present case demonstrates metastasis 25 years post-surgery, highlighting the prolonged latency of ccRCC and the challenges associated with long-term follow-up and management. This case is significant not only for its extended latency but also for the triple metastasis involving critical organs, including the rare occurrence of breast metastasis. Brain metastasis generally signifies a poorer prognosis, and such multisite, ultra-late recurrence has substantial clinical implications. This case emphasizes the importance of extending postoperative follow-up for ccRCC patients beyond 25 years to potentially improve patient outcomes.

### 3.2 Enhancing recurrence detection through molecular and imaging diagnostics

Early detection of disease recurrence through screening is essential for developing appropriate treatment strategies. Regular breast imaging examinations play a key role in the early identification of potential recurrence or metastasis, particularly during postoperative follow-up and subsequent treatment (14). Studies have demonstrated that patients who undergo regular imaging surveillance experience significantly improved survival rates, especially when active follow-up is conducted post-surgery (15). Additionally, long-term follow-up should include monitoring for metastases at other potential sites (e.g., lungs, bones), which is particularly important in patients with multiple metastases (16). Over time, tumor cells may develop metastases at new sites. Therefore, regular imaging evaluations are crucial for early detection, allowing for timely adjustments to treatment plans and ultimately improving patient quality of life and survival outcomes.

Histologically, breast metastases from ccRCC exhibit features similar to those of primary clear cell carcinoma. Metastatic lesions typically display clear cells arranged in nests or alveoli, with abundant clear cytoplasm. These characteristic cytomorphological features assist pathologists in the preliminary diagnosis and differentiation of ccRCC metastases. Immunohistochemical analysis of breast metastases typically shows positive expression of PAX8, CAIX, and CD10, while estrogen receptor (ER), progesterone receptor (PR), and HER2 are generally negative, aiding in the differentiation from primary breast cancer (5). Additionally, elevated levels of breast cancer-related tumor markers, such as CEA, CA15-3, and CA27-29, may suggest metastatic disease (17), although these markers were within normal limits in this patient. In addition to histopathological and immunohistochemical features, imaging modalities play a pivotal role in the initial evaluation of breast lesions.

Imaging plays a crucial role in the initial evaluation of breast metastases from ccRCC. Mammography and breast ultrasound are commonly employed, typically revealing ill-defined hypoechoic or high-density masses with atypical or ambiguous features for malignancy. One study noted that metastatic tumors on ultrasound often present as irregularly shaped or poorly defined masses, consistent with malignant characteristics (18). Furthermore, dynamic contrast-enhanced MRI is particularly effective in early tumor detection, demonstrating rapid enhancement and washout patterns, which assist in further differential diagnosis. Research on dynamic contrast-enhanced MRI has indicated that rapid homogeneous enhancement is a characteristic feature of metastatic tumors, providing essential information for distinguishing metastatic breast lesions from other masses (19). However, designing effective long-term follow-up for ccRCC must balance the critical need for early detection against the significant risks and cumulative burdens of intensive screening. As highlighted by Wilson et al. (2024), routine surveillance imaging after curative-intent treatment lacks evidence for improving overall survival and can impose substantial harms, including patient anxiety, radiation exposure, investigation of incidental findings, and financial toxicity (20). Therefore, a risk-adapted, stratified surveillance strategy is recommended over a uniform protocol of intensive imaging for all patients. Follow-up intensity and methods should be dynamically tailored for each individual. The patient’s initial pathological risk profile and, where available, molecular risk markers should serve as the foundation for this personalized strategy, guiding the frequency and type of monitoring.

To implement the risk-stratified surveillance strategy in practice, guidelines such as those from the European Association of Urology (EAU) can be referenced: for low-risk patients (e.g., pT1, low-grade), annual clinical examinations and basic imaging (e.g., chest X-ray) are recommend for the first 3–5 years postoperatively, followed by a transition to symptom-based monitoring. Intermediate-risk patients require more frequent assessments, such as chest and abdominal CT scans every 6 months for the first 3 years, with extended intervals thereafter. High-risk patients (e.g., pT3–4 or high-grade) need comprehensive evaluations, including chest and abdominal CT/MRI, every 3–6 months (21). However, the benefits and risks of surveillance must be balanced. Intensive follow-up incurs substantial costs (approximately $1,740-$3,700 per patient) and offers limited benefit for low-risk patients (22), while also posing risks such as radiation exposure, overtreatment due to false positives, and psychological burden (23). Therefore, evidence-based de-escalation of follow-up is crucial for long-term survivors (24).Specifically, for patients with low-risk features or those in ultra-long-term remission, clinical follow-up can be safely de-escalated to prioritize symptom-triggered evaluations over intensive imaging surveillance. For rare metastatic sites such as the brain and breast, early detection strategies should build upon effective patient education. This involves clearly informing patients about these rare events and empowering them to seek prompt medical evaluation for any new neurological symptom or discovered painless breast lump. This model effectively represents a shift from passive “routine screening” to an active, “symptom-prompted” investigation.

### 3.3 The value of combined targeted immunotherapy in ultra-late multisite metastases

Combined targeted immunotherapy (immune checkpoint inhibitors plus anti-angiogenic targeted agents) has demonstrated significant clinical value in ultra-late multisite metastatic tumors, particularly in solid malignancies such as renal cell carcinoma and hepatocellular carcinoma, offering superior survival benefits compared to conventional therapies (25, 26). In a median follow-up of 12.8 months, the pembrolizumab-axitinib group showed an estimated 12-month survival rate of 89.9%, compared to 78.3% in the sunitinib group. The median progression-free survival was 15.1 months for the pembrolizumab-axitinib group versus 11.1 months for the sunitinib group. The objective response rate was 59.3% in the pembrolizumab-axitinib group and 35.7% in the sunitinib group (27) (**Table 2**). For this patient, a combination of a PD-1 inhibitor and a tyrosine kinase inhibitor (TKI) was planned. This therapeutic rationale aligns directly with the aforementioned biological mechanisms: immune checkpoint inhibitors are designed to reactivate anti-tumor immunity, potentially targeting any residual dormant cell reservoir, while anti-angiogenic TKIs aim to sustain a state of “angiogenic suppression.” For ultra-late recurrences, such combination therapy, therefore, represents not merely an attack on visible lesions but also a systemic intervention against the permissive microenvironment that may harbor dormant disease.

**Table 2:** Efficacy of pembrolizumab + axitinib vs. sunitinib in the treatment of advanced renal cell carcinoma (KEYNOTE-426) (27).

Evaluation indicators	Pembrolizumab + Axitinib group	Sunitinib group	Statistical difference
12-month OS	89.9%	78.3%	P<0.0001
mPFS	15. 1 month	11.1 month	P<0.001
ORR	59.3%	35.7%	P<0.001

OS, overall survival rate; mPFS, median progression-free survival; ORR, objective response rate.

## 4 Clinical implications

The present case offers several critical takeaways for clinical practice:

First, regarding long-term surveillance, this ultra-late recurrence suggests that clear cell renal cell carcinoma can maintain a dormant state for decades before progressing. This challenges the conventional 5-10-year follow-up paradigm and strongly advocates for lifelong, albeit risk-adapted, follow-up strategies in RCC patients, considering it a chronic disease with lifelong metastatic potential.

Second, regarding diagnosis, this case provides a clear diagnostic roadmap for the evaluating of a breast mass in a patient with a prior malignancy. It underscores the necessity of a high index of suspicion and the pivotal role of multi-modality imaging (mammography, ultrasound, MRI) coupled with confirmatory biopsy and immunohistochemical profiling (e.g., PAX8, CAIX, CD10) to reliably distinguish metastatic RCC from primary breast cancer, thereby avoiding misdiagnosis and guiding appropriate management.

Finally, regarding therapeutic approach, the emergence of multi-metastatic disease in this treatment-naïve elderly patient, while challenging, now has promising therapeutic options. This case illustrates the modern shift away from cytokines and pure VEGF inhibition towards combination immunotherapy and targeted therapy (e.g., immune checkpoint inhibitors paired with TKIs), which have demonstrated superior efficacy in advanced RCC and offer a potent strategy even for ultra-late recurrences.

## 5 Conclusion

In summary, although breast metastasis from ccRCC is exceptionally rare, the extraordinarily long metastatic latency period of up to 25 years further expands our understanding; heightened awareness of this phenomenon should encourage clinicians to carefully monitor patients with relevant medical histories for the development of new lesions. This proactive approach not only facilitates early diagnosis but may also enhance patient prognosis.

## Data Availability

The original contributions presented in the study are included in the article/supplementary material. Further inquiries can be directed to the corresponding author.
